# Stroke in HIV-infected individuals with and without HCV coinfection in Spain in the combination antiretroviral therapy era

**DOI:** 10.1371/journal.pone.0179493

**Published:** 2017-06-15

**Authors:** Alejandro Alvaro-Meca, Juan Berenguer, Asunción Díaz, Dariela Micheloud, Teresa Aldámiz-Echevarría, Chiara Fanciulli, Salvador Resino

**Affiliations:** 1 Unidad de Medicina Preventiva y Salud Pública, Facultad de Ciencias de la Salud, Universidad Rey Juan Carlos, Alcorcón, Madrid, Spain; 2 Unidad de Enfermedades Infecciosas/VIH, Hospital General Universitario Gregorio Marañón, Madrid, Spain; 3 Instituto de Investigación Sanitaria Gregorio Marañón (IiSGM), Madrid, Spain; 4 Área de Vigilancia Epidemiológica de VIH/SIDA y comportamientos de riesgo, Centro Nacional de Epidemiología, Instituto de Salud Carlos III, Madrid, Spain; 5 Centro de Investigación Biomédica en Red de Epidemiología y Salud Pública (CIBERESP), Madrid, Spain; 6 Servicio de Medicina Interna, Hospital General Universitario Gregorio Marañón, Madrid, Spain; 7 Unidad de Infección Viral e Inmunidad, Centro Nacional de Microbiología, Instituto de Salud Carlos III, Majadahonda, Madrid, Spain; University of New South Wales, AUSTRALIA

## Abstract

The incidence of stroke in human immunodeficiency virus (HIV)–infected individuals has been well analyzed in recent epidemiological studies. However, little is known about the specific contribution of hepatitis C virus (HCV) infection to stroke among HIV-infected individuals. The aims of this study were to analyze trends in the incidence rates of stroke in HIV-infected individuals during the combination antiretroviral (cART) era in Spain and to categorize them by the presence or absence of HCV coinfection. We analyzed hospital discharges with a diagnosis of stroke in Spain according to ICD-9-CM during 1997–2013. The study period was divided into four calendar periods (1997–1999, 2000–2003, 2004–2007, and 2008–2013). Patients were classified according to HCV serology. The number of HIV-infected patients was estimated based on data from the National Centre of Epidemiology. We calculated incidence rates (events per 10,000 patient-years) and in-hospital case fatality rates (CFR). The incidence of hemorrhagic stroke (HS) decreased in HIV-monoinfected patients (15.8 [1997–1999] to 6.5 [2008–2013]; *P*<0.001) and increased in HIV/HCV-coinfected patients (1.3 [1997–1999] to 5.5 [2008–2013]; *P*<0.001). The incidence of ischemic stroke (IS) decreased in HIV-monoinfected patients (27.4 [1997–1999] to 21.7 [2008–2013]; *P* = 0.005) and increased in HIV/HCV-coinfected patients (1.8 [1997–1999] to 11.9 [2008–2013]; *P*<0.001). The CFR was 3.3 times higher for HS than for IS for the whole study period. The CFR of HS in HIV-monoinfected patients decreased significantly (47.4% [1997–1999] to 30.6% [2008–2013]; *P* = 0.010) but did not change significantly among HIV/HCV-coinfected patients (41.4% [1997–1999] to 44.7% [2008–2013]; *P* = 0.784). The CFR of IS in the whole HIV-infected population decreased significantly (14.6% [1997–1999] to 10.9% [2008–2013]; *P* = 0.034), although no significant differences were found when each group was analyzed separately. In conclusion, after the introduction of cART, HS and IS rates decreased in HIV-monoinfected individuals, but increased steadily in HIV/HCV-coinfected individuals.

## Introduction

Combination antiretroviral therapy (cART) has significantly improved the life expectancy of human immunodeficiency virus (HIV)–infected individuals [1]. The implications of this improvement have proven substantial, since up to 30% of adults living with HIV in high-income countries today are aged 50 years or over [2], and most of those on cART will be above this age in 2030 [3]. Consequently, treatment and prevention of aging-related conditions including dyslipidemia and other metabolic disorders, cardiovascular diseases, non–AIDS-related cancers, kidney disease, osteoporosis, and neurocognitive disorders has become a major issue in the management of HIV-infected individuals [4].

Stroke is one of the most important health problems worldwide, particularly in middle- and low-income countries [5]. Most of the recognized risk factors for stroke such as hypertension, aging, diabetes mellitus, hypercholesterolemia, and smoking are essentially cardiovascular risk factors [6]. However, infectious agents such as HIV have been found to increase the risk of stroke [7]. HIV infection can increase the risk of stroke indirectly through opportunistic infections, cancer, cardioembolic stroke, and coagulopathy or directly through HIV-associated vasculopathy [8]. In addition, cART may contribute directly to stroke by accelerating atherosclerosis or indirectly by increasing life expectancy and thus exposure to conventional vascular risk factors [8].

The incidence of stroke in HIV-infected individuals and differences in the incidence of stroke between HIV-infected and non–HIV-infected individuals were well analyzed in a recent epidemiological study [9]. However, little is known about the specific contribution of hepatitis C virus (HCV) infection to stroke among HIV-infected individuals. This observation is interesting, because HCV infection, one of the most common comorbid conditions in HIV-infected patients [10], has been found to increase the risk of stroke [11]. The aims of this study were to analyze trends in the incidence rates of stroke in HIV-infected individuals during the cART era in Spain and to categorize them by the presence or absence of HCV coinfection.

## Materials and methods

### Study period and population

We performed a retrospective analysis of HIV-infected individuals aged over 15 years who were discharged from Spanish hospitals with a diagnosis of stroke from 1 January 1997 to 31 December 2013. We subdivided the study period into four calendar periods according to the widespread use of cART among HIV-infected individuals [12], as follows: a) 1997–1999; b) 2000–2003; c) 2004–2007; and d) 2008–2013.

Data were obtained from the Spanish Minimum Basic Data Set (CMBD, *Conjunto Mínimo Básico de Datos*), which is provided by the Ministry of Health, Social Services, and Equality (MSSSI). The CMBD is a clinical and administrative database that records information on hospital discharges in Spain. It was established in 1987 and has an estimated coverage of 97.7% of all admissions to public hospitals [13]. The CMBD provides the encrypted patient identification number, sex, date of birth, dates of hospital admission and discharge, medical institutions providing the services, diagnosis and procedure codes according to the *International Classification of Diseases*, *9th ed*, *Clinical Modification* (ICD-9-CM), and outcome at discharge [14]. According to an audit conducted in 2011, the quality of CMBD data for cerebrovascular disease is appropriate for epidemiological studies [15]. A hospitalization was defined as a discharge record in the CMBD.

### Ethics statement

This study involves the use of patients’ medical data from the Spanish CMBD, which is hosted by the MSSSI. The CMBD is regulated by legislation that stipulates how institutions are required to utilize health-related personal data. As described in detail previously [16], full data confidentiality was guaranteed according to current Spanish legislation. We requested the databases by completing, signing, and sending a questionnaire with a Confidentiality Commitment. The MSSSI evaluated our study protocol and considered that it met the appropriate ethical requirements according to Spanish legislation. Given the anonymous and mandatory nature of the dataset, it was not necessary to obtain informed consent. Additionally, our study was approved by the Research Ethics Committee (Comité de Ética de la Investigación y de Bienestar Animal) of the Instituto de Salud Carlos III (Madrid, Spain).

### ICD-9-CM codes and study groups

We selected patients who were coded in the CMBD with a diagnosis of stroke (ICD-9 430–437), which was in turn classified as hemorrhagic stroke (ICD-9 430–432) or ischemic stroke (ICD-9 433–437). ICD-9-CM codes were also used to define viral infection status: HIV infection (042 or V08), HCV infection (ICD-9-CM codes 070.44, 070.54, 070.7x, or V02.62), and hepatitis B virus (HBV) infection (ICD-9-CM codes 070.2x, 070.3x, or V02.61) (Table A in S1 File). We therefore categorized patients as HIV-monoinfected (patients solely infected with HIV) and HIV/HCV-coinfected (patients coinfected with HIV and HCV). HBV infection was a criterion for exclusion. The index episode was defined as a hospital discharge with a diagnosis of stroke. Patients who were readmitted with a diagnosis of stroke were not counted as new episodes of stroke. We also analyzed in-hospital death among patients diagnosed with stroke.

The Charlson comorbidity index, a well validated combined age-comorbidity score that is applied to estimate the relative risk of death from prognostic clinical covariates, was also calculated based on ICD-9-CM diagnoses codes [17] (Table A in S1 File).

### Reference populations

Estimates of people living with HIV/AIDS in Spain were provided by the National Centre of Epidemiology (Instituto de Salud Carlos III, Madrid, Spain) [18]. This estimation was made using the Estimation and Projection Package (EPP) and Spectrum software, both of which are used by the Joint United Nations Programme on HIV/AIDS (UNAIDS)/WHO for estimating and projecting HIV prevalence levels in countries with concentrated epidemics [19, 20].

The number of HIV-monoinfected and HIV/HCV-coinfected individuals in Spain was estimated using the results of the hospital survey of HIV/AIDS-infected patients, a second-generation surveillance system in people living with HIV coordinated by the National Centre of Epidemiology [21]. We also used reports from two Spanish national cohorts: the “Grupo de Estudio de Sida” (GeSIDA) [10] and the “Asociación Médica VACH de Estudios Multicentricos (AMVACH)” [22] (Table B in S1 File).

### Statistical analysis

We estimated incidence rates of hemorrhagic and ischemic stroke in HIV-monoinfected and HIV/HCV-coinfected individuals. The number of events within each calendar period was used as the numerator. The denominator was the number of persons at risk within each calendar period. The case fatality rate was estimated as the number of stroke-related deaths as a proportion of the number of hospitalized patients with a diagnosis of stroke. Categorical data and proportions were analyzed using the chi-squared test or Fisher exact test, as required. The *t* test or Mann-Whitney test was used to compare continuous variables. Temporal trends in incidence rates of stroke were evaluated using the Extended Mantel-Haenszel chi-square test for linear trend. We also calculated the odds of in-hospital death in patients with a diagnosis of stroke according to HIV and HCV status using logistic regression models, which were adjusted for age, sex, tobacco use, and Charlson comorbidity index [17].

The statistical analysis was performed using the statistical package R, version 3.1.1 (GNU General Public License) [23]. All tests were two-tailed, and p values <0.05 were considered significant. A Bonferroni correction was used to adjust statistical significance for multiple comparisons.

## Results

### Characteristics of the study population

We identified a total of 1,763,259 patients discharged from Spanish hospitals with a diagnosis of stroke from 1 January 1997 to 31 December 2013. Of these, 4,091 (0.23%) were infected with HIV (2,853 HIV-monoinfected and 1238 HIV/HCV-coinfected). The main characteristics of HIV-infected individuals categorized by the presence or absence of HCV coinfection are shown in Table 1.

**Table 1:** Epidemiological and clinical characteristics of HIV-infected patients with a hospital admission and diagnosis of stroke from 1997 to 2013 in Spain.

	All patients	HIV-monoinfected	HIV/HCV-coinfected	P*
**No. of patients**	4091	2853	1238	
**Gender (male), No. (%)**	3250 (79.4%)	2259 (79.2%)	991 (80%)	0.556
**Age (years), median (IQR)**	45 (15)	46 (20)	44 (10)	<0.001
**Arterial hypertension, No. (%)**	708 (17.3)	539 (18.9)	169 (13.7)	<0.001
**Diabetes mellitus without complications, No. (%)**	366 (8.9)	281 (9.8)	85 (6.9)	0.003
**Diabetes mellitus with complications, No. (%)**	52 (1.3)	44 (1.5)	8 (0.6)	0.028
**Renal disease, No. (%)**	195 (4.8)	133 (4.7)	62 (5)	0.691
**Charlson comorbidity index, median (IQR)**	1 (2)	1 (2)	1 (2)	0.051
**Substance use around admission date, No. (%)**				
Illicit drugs	1844 (45.1)	1063 (37.3)	781 (63.1)	<0.001
Alcohol	151 (3.7)	74 (2.6)	77 (6.2)	<0.001
Tobacco	1093 (26.7)	669 (23.4)	424 (34.2)	<0.001
**Conditions influencing health status, No. (%)**				
Surgical conditions (V42, V45)	161 (3.9)	119 (4.2)	42 (3.4)	0.276
Trauma (E880* to E929*, E950 to E999*)	109 (2.7)	67 (2.3)	42 (3.4)	0.072
**Diagnoses related to stroke, No. (%)**				
**Hemorrhagic stroke** (ICD-9 430–432)	1272 (31.1)	802 (28.1)	470 (38)	<0.001
Subarachnoid hemorrhage (ICD-9 430)	280 (6.8)	186 (6.5)	94 (7.6)	0.237
Intracerebral hemorrhage (ICD-9 431)	756 (18.5)	459 (16.1)	297 (24)	<0.001
Other and unspecified intracranial hemorrhage (ICD-9 432)	288 (7)	191 (6.7)	97 (7.8)	0.214
**Ischemic stroke** (ICD-9 433–437)	2923 (71.4)	2122 (74.4)	801 (64.7)	<0.001
Occlusion and stenosis of precerebral arteries (ICD-9 433)	385 (9.4)	309 (10.8)	76 (6.1)	<0.001
Occlusion of cerebral arteries (ICD-9 434)	1476 (36.1)	1098 (38.5)	378 (30.5)	<0.001
Transient cerebral ischemia (ICD-9 435)	319 (7.8)	231 (8.1)	88 (7.1)	0.308
Acute but ill-defined cerebrovascular disease (ICD-9 436)	159 (3.9)	121 (4.2)	38 (3.1)	0.090
Other and ill-defined cerebrovascular disease (ICD-9 437)	816 (19.9)	540 (18.9)	276 (22.3)	0.015
**Hemorrhagic-to-ischemic stroke ratio**				
Whole period	0.4	0.4	0.6	<0.01
1997–1999	0.6	0.6	0.7	0.35
2000–2003	0.5	0.4	0.7	<0.01
2004–2007	0.5	0.4	0.8	<0.01
2008–2013	0.4	0.3	0.5	<0.001
**Length of hospital stay (days), median (IQR)**	11 (16)	11 (16)	10 (14)	<0.001
**Sequelae of stroke, No. (%)**				
Late effects of cerebrovascular disease (ICD-9 438)	122 (3)	85 (3)	37 (3)	0.999

*P values, refer to the differences between HIV-monoinfected and HIV/HCV-coinfected, and were calculated using the chi-square test and Mann-Whitney test.

Abbreviations: HCV, hepatitis C virus; HIV, human immunodeficiency virus.

In brief, compared with HIV-monoinfected patients, HIV/HCV-coinfected patients were younger and had a lower frequency of arterial hypertension and diabetes mellitus, a higher frequency of substance abuse (illicit drugs, alcohol, and tobacco), more hemorrhagic stroke–related diagnoses and fewer ischemic stroke–related diagnoses, and a higher hemorrhagic-to-ischemic stroke ratio. Stroke-related sequelae were reported with equal frequency in both groups.

### Incidence of stroke

**Fig 1:**
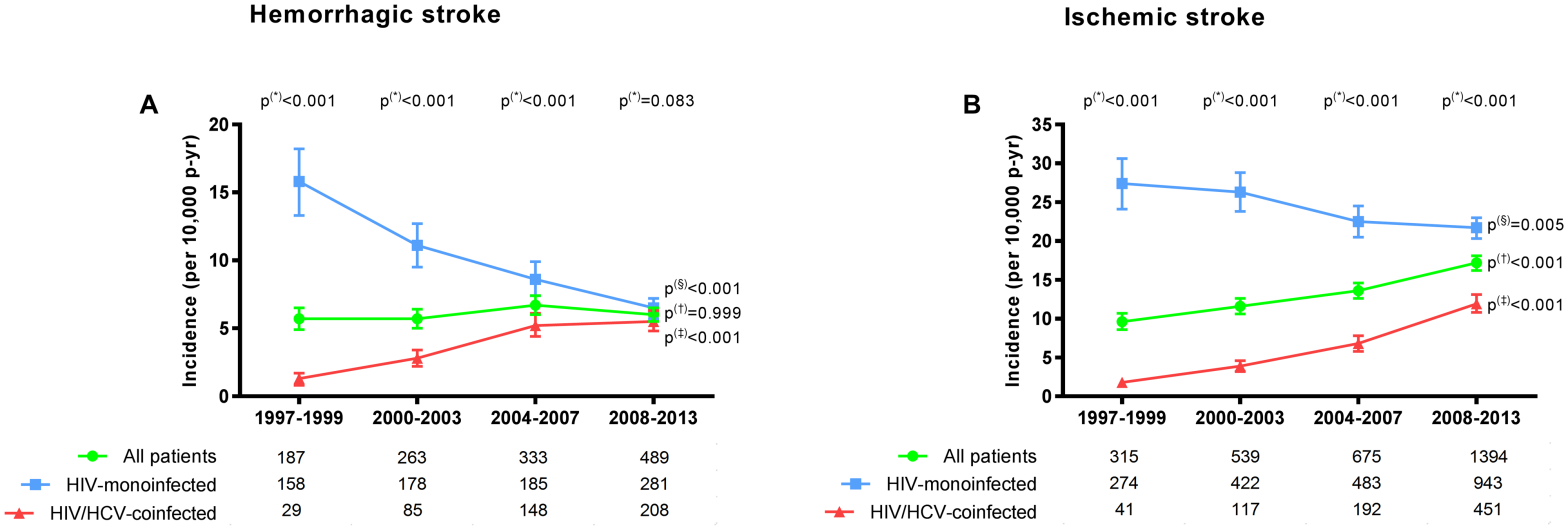
Incidence rates of hospitalization for hemorrhagic stroke (Fig 1A) and for ischemic stroke (Fig 1B) in the four study periods. P-values: (*), differences between HIV-monoinfected patients and HIV/HCV-coinfected patients by the exact confidence intervals for incidence; (§), linear trend from 1997–1999 to 2008–2013 in HIV-monoinfected patients by the Extended Mantel-Haenszel chi-square; (†), linear trend from 1997–1999 to 2008–2013 in HIV-infected patients by the Extended Mantel-Haenszel chi-square; (‡), linear trend from 1997–1999 to 2008–2013 in HIV/HCV-coinfected patients by the Extended Mantel-Haenszel chi-square. The numbers at the base of the panels are the total numbers of hospitalizations in each calendar period and study group. Abbreviations: HCV, hepatitis C virus; HIV, human immunodeficiency virus; p-yr, patient-years.

The incidence rates of hospitalization for hemorrhagic stroke (events per 10,000 patient-years) in the four study periods are shown in Fig 1A (full description in Table C in S1 File).

In brief, the incidence rates of hospitalization for hemorrhagic stroke remained stable in HIV-infected patients throughout the study period. When the population was stratified by HCV status, the rate of hospitalization for hemorrhagic stroke was significantly higher in HIV-monoinfected patients than in HIV/HCV-coinfected patients in the early cART period (15.8 vs. 1.3; p<0.001). However, from the early to the late cART periods, the incidence of hospitalization for hemorrhagic stroke decreased significantly in HIV-monoinfected patients (from 15.8 to 6.5; p<0.001), whereas it increased significantly in HIV/HCV-coinfected patients (from 1.3 to 5.5; p<0.001). In the late cART period, rates of hospitalization for hemorrhagic stroke in HIV/HCV-coinfected patients and in HIV-monoinfected patients were similar (6.5 and 5.5, respectively; p = 0.083).

The incidence rates of hospitalization for ischemic stroke (events per 10,000 patient-years) in the four study periods are shown in Fig 1B (full description in Table C in S1 File). In brief, in HIV-infected patients, the incidence of hospitalization for ischemic stroke increased significantly throughout the study period (from 9.6 to 17.2; p<0.001). When the population was stratified by HCV status, the rate of hospitalization for ischemic stroke was higher in HIV-monoinfected patients than in HIV/HCV-coinfected patients in the early cART period (27.4 vs. 1.8; p<0.001). However, from the early cART period to the late cART period, the rate of hospitalization for ischemic stroke decreased significantly in HIV-monoinfected patients (from 27.4 to 21.7; p = 0.005) but increased significantly in HIV/HCV-coinfected patients (from 1.8 to 11.9; p<0.001). In the late cART period, rates of hospitalization for ischemic stroke were higher in HIV-monoinfected patients than in HIV/HCV-coinfected patients (21.7 vs. 11.9; p<0.001).

**Fig 2:**
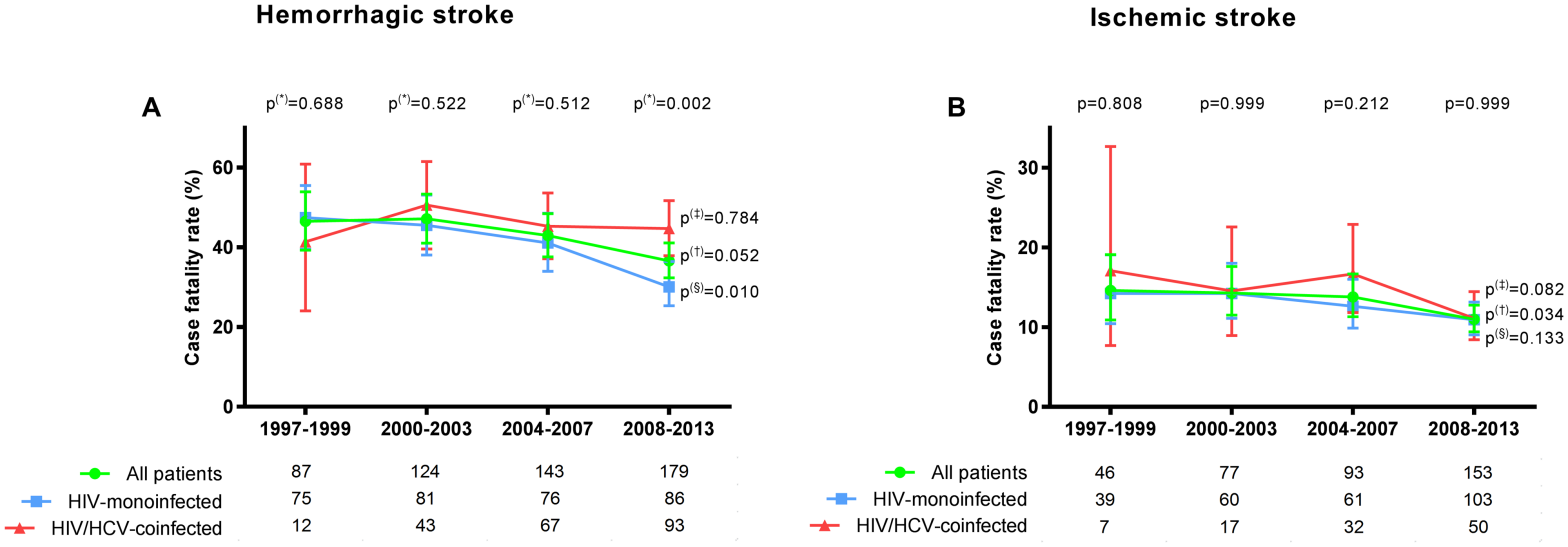
Case fatality rates for hemorrhagic stroke (Fig 2A) and for ischemic stroke (Fig 2B) in the four study periods stratified by HCV status. P-values: (*), differences between HIV-monoinfected patients and HIV/HCV-coinfected patients by the exact confidence intervals for incidence; (§), linear trend from 1997–1999 to 2008–2013 in HIV-monoinfected patients by the Extended Mantel-Haenszel chi-square; (†), linear trend from 1997–1999 to 2008–2013 in HIV-infected patients by the Extended Mantel-Haenszel chi-square; (‡), linear trend from 1997–1999 to 2008–2013 in HIV/HCV-coinfected patients by the Extended Mantel-Haenszel chi-square. The numbers at the base of the panels are the total numbers of deaths in each calendar period and study group. Abbreviations: HCV, hepatitis C virus; HIV, human immunodeficiency virus.

Case fatality rates for hemorrhagic and ischemic stroke are shown in Fig 2 (full description in Table D in S1 File).

The case fatality rate was 3.3 times higher for hemorrhagic stroke than for ischemic stroke throughout the study period and varied 3.1 to 3.4–fold in the four subperiods. The case fatality rate of hemorrhagic stroke in HIV-monoinfected patients decreased significantly from 47.4% in the period 1997–1999 to 30.6% in the period 2008–2013 (p = 0.010) but did not change significantly among HIV/HCV-coinfected patients during the study period: 41.4% in the period 1997–1999 and 44.7% in the period 2008–2013 (p = 0.784). Consequently, the case fatality rate for hemorrhagic stroke was significantly lower in HIV-monoinfected patients than in HIV/HCV-coinfected patients in the late cART period (30.6% vs 44.7%; p = 0.002). The case fatality rate of ischemic stroke in the whole HIV-infected population decreased significantly from 14.6% in the period 1997–1999 to 10.9% in the period 2008–2013 (p = 0.034); however, no significant differences were found when each group was analyzed separately.

**Fig 3:**
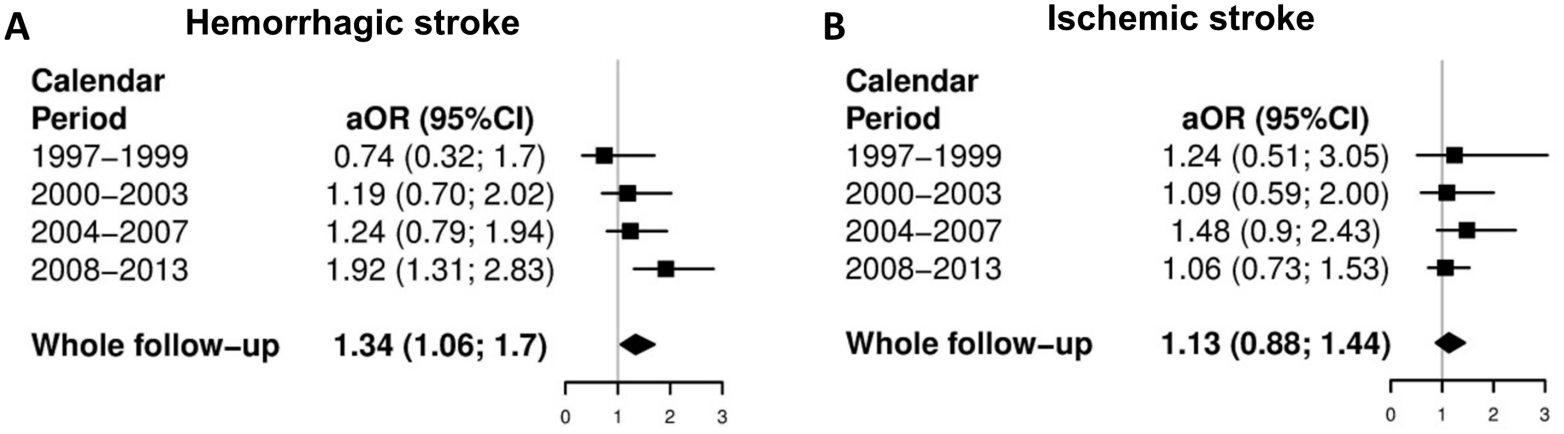
Adjusted likelihood of death from hemorrhagic and ischemic stroke in HIV/HCV-coinfected patients compared with HIV-monoinfected patients in Spain (1997–2013). Abbreviations: HCV, hepatitis C virus; HIV, human immunodeficiency virus; aOR, adjusted odds ratio; 95%CI, 95% confidence interval.

Logistic regression analysis showed that the adjusted odds ratio of death following hemorrhagic stroke was significantly higher in HIV/HCV-coinfected patients than in HIV-monoinfected patients in the late cART period (Fig 3A). However, the adjusted odds of death following ischemic stroke were not significantly different between HIV/HCV-coinfected patients and HIV-monoinfected patients during the study period (Fig 3B).

For a full comparison of clinical characteristics, rates of stroke, and mortality rates between the non–HIV-infected and the HIV-infected populations in Spain in the study period (1997–2013), see Tables D,E, and F in S1 File and Figure A in S1 File.

## Discussion

We carried out a retrospective study of adults discharged from Spanish hospitals with a diagnosis of stroke during the first 17 years after the introduction of cART. We found that in the early cART period (1997–1999), the incidence rates of hemorrhagic and ischemic stroke were significantly higher for HIV-monoinfected patients than for HIV/HCV-coinfected patients. However, during the following years, the incidence rates of both types of stroke decreased in the former group and increased in the latter. The net result was that in the last cART period (2008–2013), the incidence rate of hospitalization for hemorrhagic stroke was similar for both groups of patients. However, incidence rates of hospitalization for ischemic stroke remained higher for HIV-monoinfected patients than for HIV/HCV-coinfected patients. To our knowledge, this is the first study to assess temporal trends of incidence rates of stroke in HIV-infected patients who were stratified by HCV serostatus.

A noteworthy finding in our study was the statistically significant decline in incidence rates for both types of hospitalization in HIV-monoinfected patients. Several factors could have influenced these findings. First, the proportion of known HIV-infected individuals receiving cART in Spain has increased significantly over the last decade, and more patients have a fully suppressed HIV viral load, a less advanced CDC clinical stage, and higher CD4+ T-cell counts [21]. These factors are important, because the results of a randomized clinical trial show that continuous suppression of HIV with cART reduces not only the risk of new AIDS-defining conditions and death, but also the risk of non–AIDS-defining conditions including cardiovascular events [24]. In addition, observational studies have shown that high plasma HIV viral load and/or low CD4+ T-cell counts increase the risk of stroke in HIV-infected individuals [8, 25–27]. The second factor is the recognition by clinicians of the importance of cardiovascular disease in HIV-infected individuals after the introduction of cART, together with improvements in preventive interventions to reduce cardiovascular risk, which have resulted in a steady reduction in cardiovascular mortality in high-income countries [28]. Finally, the introduction of drugs with fewer metabolic side effects could have contributed to the decrease in the incidence of stroke.

Another remarkable finding was the significant increase in the incidence rates of hospitalization for stroke in HIV/HCV-coinfected patients. At the beginning of the study period, the incidence rates of both types of stroke were significantly lower in coinfected patients than in HIV-monoinfected patients; however, in the following years, the incidence rates of stroke increased significantly in coinfected patients. At the end of the study period, rates of hemorrhagic stroke were similar in both patient groups, although rates of ischemic stroke were still lower for coinfected patients than for monoinfected patients. We were unable to identify factors that could explain the increasing rates of hospitalization in coinfected individuals, for several reasons. First, there may have been differences in testing for HCV infection in patients admitted to hospitals with stroke (e.g., more HCV testing among HIV-positive patients in the late period than during the early period). However, this is unlikely, because the proportion of HIV-infected patients tested for HCV antibodies in Spain according to prevalence studies has not changed significantly over the last 13 years and has always been over 95% [10, 29]. Second, we believe it is much more likely that death was a major competing risk for stroke in coinfected patients during the first years after the introduction of cART but that its relevance waned during the remainder of the study period. This possibility seems to be justified by the observation that although mortality trends for HIV-infected individuals in Spain declined significantly when the first years of the cART era are taken as the reference, this was mostly at the expense of HIV-monoinfected patients, since declines in mortality rates were not observed among HIV/HCV-coinfected patients [30]. It is important to mention that injection drug use has been—and remains so today—the predominant mode of HIV acquisition in patients with HCV antibodies in Spain [10, 21, 29]. In other words, these patients are not only at risk of AIDS-related and liver-related deaths, but they are also at risk of death related to lifestyle factors including substance use, accidents, and suicide [10]. Although outbreaks of HCV infection among men who have sex with men who engage in high-risk sexual practices have been reported in some hospitals in Madrid and Barcelona [31, 32], sexually acquired HCV infection has never contributed significantly to the burden of HIV/HCV coinfection in Spain [10]. In the last years of the study, several factors could have contributed to decreasing mortality rates among coinfected patients and, consequently, to the decrease in the competing risk of death. These include the use of simpler, more efficacious and safer cART regimens for all clinical scenarios [33], and the increasing uptake of anti-HCV therapy by coinfected patients [10]. Both factors are associated with reductions in liver-related and non–liver-related mortality among coinfected individuals [34–37].

Patients with hemorrhagic stroke are generally at a higher risk of death than patients with ischemic stroke [38], as shown in the present study, where the case fatality rate was 3.3 times higher for hemorrhagic stroke than for ischemic stroke. We also found that the case fatality rates for stroke decreased during the study period, except for hemorrhagic stroke in HIV/HCV-coinfected patients, in whom a non-significant increase was observed in the case fatality rate. Unfortunately, we have no information about therapies or procedures that might have influenced survival.

This study has several limitations. First, we did not have verified data for persons infected with HIV in Spain from 1997 to 2013, because there were no national coverage data for HIV diagnoses during this period. Instead, we used an estimate provided by the National Centre of Epidemiology [18]. Furthermore, by working with estimated populations, we were unable to calculate the incidence stratified by age and gender. However, it should be noted that the mean age of both study groups was quite similar (46 vs. 44). Second, we do not have information about how many patients are likely to have had a stroke and died before admission to hospital. However, there are no reasons to suspect that this may have changed during the study period. Third, owing to the nature of our database, we had no access to key clinical data that could have helped us to interpret our results more completely. Such information includes reliable data on illicit drug use, cART regimens and adherence, CD4+ cell counts, HIV viral loads, stroke management, and management of comorbidities such as hypertension and diabetes. Although illicit drug use was more frequently reported by HIV/HCV-coinfected patients than by HIV-monoinfected patients, we lacked reliable information about the use of cocaine and amphetamines, both of which are known risk factors for stroke [39]. In the case of cocaine, the frequency of intracranial hemorrhage exceeds that of cerebral infarction [40]. It is thus plausible, that illicit drug use could have contributed to increasing rates of hemorrhagic stroke among HIV/HCV-coinfected patients during the study periods. Finally, coinfection by HCV was defined by the presence of anti-HCV antibodies, and we were unable to estimate the proportion of patients with active HCV infection defined by the presence of HCV-RNA in serum. This problem is common in cohort studies [41, 42] and meta-analyses [43].

The last limitation raises the question of whether HCV *per se* (not considering lifestyle factors) contributes to atherosclerosis and cardiovascular disease. From a pathophysiological perspective, HCV infection is associated with increased levels of inflammatory markers and endothelial dysfunction, which could potentially stimulate atherosclerosis [44–46]. However, HCV infection is also associated with lower serum levels of total cholesterol and LDL cholesterol, which is clearly a protective factor against atherosclerosis [47, 48]. From a clinical perspective, although HCV infection has been found to increase the likelihood of cardiovascular disease among HIV-infected individuals in some observational studies [49–51] and among the general population in other studies [52–60], no association has been found between HCV infection and angiography-proven coronary artery disease [61, 62] or myocardial infarction [63]. As for meta-analyses, an association between HCV infection and an increased risk of cardiovascular events (including stroke) has been found in some studies [11, 64], but not in others [65]. Finally, eradication of HCV may have different effects on atherogenesis: reversion of inflammation and endothelial dysfunction [66] and rebound of LDL and total cholesterol to levels associated with increased risk of coronary artery disease [47, 67].

## Conclusions

In conclusion, we used a large and well-coded administrative database encompassing the vast majority of admissions to public hospitals in Spain to study the incidence of stroke in HIV-infected patients over a long period. We found that the hemorrhagic-to-ischemic stroke ratio was higher in HIV/HCV-coinfected patients than in HIV-monoinfected patients. We also found contrasting trends in the incidence of stroke according to HCV serostatus, namely, decreasing trends in HIV-monoinfected patients and increasing trends in HIV/HCV-coinfected patients. The in-hospital case fatality rate was more than three times higher for hemorrhagic stroke than for ischemic stroke, and—with the exception of hemorrhagic stroke in HIV/HCV-coinfected patients—the in-hospital case fatality rates for stroke decreased during the study period. Our findings suggest that stroke is an emerging health problem among HIV/HCV-coinfected patients. Further studies should be designed to corroborate these findings, to ascertain the contribution of lifestyle factors and active HCV infection to stroke, and to assess the possible impact of new anti-HCV therapies for reducing the risk of stroke in coinfected patients. Finally, we wish to stress that despite the differences found in the incidence of stroke among HIV-monoinfected and HIV/HCV-coinfected individuals, the absolute numbers of stroke among both groups increased steadily during the study period. This finding, most likely due to the increasing number of older people living with HIV, emphasizes that stroke is increasingly frequent among HIV-infected individuals.

## Supporting information

S1 File Table A. Summary of ICD-9-CM coding used in this study. Table B. Estimation of the number of people aged over 15 years coinfected with HIV and HCV in Spain. Table C. Incidence of stroke diagnosed (events per 10,000 person-years) in Spain (1997–2013) stratified by calendar period. Table D. Case fatality rate of stroke (percentage) in Spain (1997–2013) stratified by calendar period. Table E. Epidemiological and clinical characteristics of patients with a hospital admission and diagnosis of stroke from 1997 to 2013 in Spain: non–HIV-infected vs HIV-infected. Table F. Incidence of stroke diagnosed (events per 10,000 person-years) in Spain (1997–2013) stratified by calendar period: non–HIV-infected vs HIV-infected. Table G. Case fatality rate of stroke (percentage) in Spain (1997–2013) stratified by calendar period: non–HIV-infected vs HIV-infected. Figure A. Adjusted odds of death due to hemorrhagic and ischemic stroke in Spain (1997–2013) in HIV-infected patients in comparison with non–HIV-infected patients.
(DOCX)
