# Supplementary material for: Stroke in HIV-infected individuals with and without HCV coinfection in Spain in the combination antiretroviral therapy era
Source: PLoS One. 2017 Jun 15;12(6):e0179493. doi: 10.1371/journal.pone.0179493 (PMC5472313; doi:10.1371/journal.pone.0179493)
Supplement: S1 File — Table A. Summary of ICD-9-CM coding used in this study. Table B. Estimation of the number of people aged over 15 years coinfected with HIV and HCV in Spain. Table C. Incidence of stroke diagnosed (events per 10,000 person-years) in Spain (1997–2013) stratified by calendar period. Table D. Case fatality rate of stroke (percentage) in Spain (1997–2013) stratified by calendar period. Table E. Epidemiological and clinical characteristics of patients with a hospital admission and diagnosis of stroke from 1997 to 2013 in Spain: non–HIV-infected vs HIV-infected. Table F. Incidence of stroke diagnosed (events per 10,000 person-years) in Spain (1997–2013) stratified by calendar period: non–HIV-infected vs HIV-infected. Table G. Case fatality rate of stroke (percentage) in Spain (1997–2013) stratified by calendar period: non–HIV-infected vs HIV-infected. Figure A. Adjusted odds of death due to hemorrhagic and ischemic stroke in Spain (1997–2013) in HIV-infected patients in comparison with non–HIV-infected patients. (DOCX) [file pone.0179493.s001.docx]

Table A. **Summary of ICD-9-CM coding used in this study**.

| **Description** | **ICD-9-CM** |
| --- | --- |
| **Diagnoses related to stroke** |  |
| **Hemorrhagic stroke** | 430-432 |
| Subarachnoid hemorrhage | 430 |
| Intracerebral hemorrhage | 431 |
| Other and unspecified intracranial hemorrhage | 432 |
| **Ischemic stroke** | 433-437 |
| Occlusion and stenosis of precerebral arteries | 433 |
| Occlusion of cerebral arteries | 434 |
| Transient cerebral ischemia (ICD-9) | 435 |
| Acute but ill-defined cerebrovascular disease | 436 |
| Other and ill-defined cerebrovascular disease | 437 |
| **Sequelae of stroke** |  |
| Late effects of cerebrovascular disease | 438 |
| **Viral infection status** |  |
| HIV infection | 042 or V08 |
| HCV infection | 070.44, 070.54, 070.7x, or V02.62 |
| HBV infection | 070.2x, 070.3x, or V02.61 |
| **Conditions affecting health status** |  |
| Surgical conditions | V42, V45 |
| Trauma | E880* to E929*, E950 to E999* |
| **Comorbid diseases (Charlson index)** |  |
| Myocardial infarction | 410, 412 |
| Congestive heart failure | 398.91, 402.01, 402.11, 402.91, 404.01, 404.03, 404.11, 404.13, 404.91, 404.93, 425.4, 425.5, 425.7, 425.8, 425.9, 428 |
| Peripheral vascular disease | 093.0, 437.3, 440, 441, 443.1, 443.2, 443.8, 443.9, 447.1, 557.1, 557.9, V434 |
| Essential hypertension | 401 |
| Cerebrovascular disease | 362.34, 430, 431, 432, 433, 434, 435, 436, 437, 438 |
| Chronic pulmonary disease | 416.8, 416.9, 490, 491, 492, 493, 494, 495, 496, 500, 501, 502, 503, 504, 505, 5064, 508.1, 508.8 |
| Connective tissue disease-rheumatic disease | 446.5, 710.0, 710.1, 710.2, 710.3, 710.4, 714.0, 714.1, 714.2, 714.8, 725 |
| Mild liver disease | 070.22, 070.23, 070.32, 070.33, 070.44, 070.54, 070.6, 070.9, 570, 571, 573.3, 573.4, 573.8, 573.9, V427 |
| Moderate or severe liver disease | 456.0, 456.1, 456.2, 572.2, 572.3, 572.4, 572.8 |
| Diabetes without complications | 250.0, 250.1, 250.2, 250.3, 250.8, 250.9 |
| Diabetes with complications | 250.4, 250.5, 250.6, 250.7 |
| Renal disease | 403.01, 403.11, 403.91, 404.02, 404.03, 404.12, 404.13, 404.92, 404.93, 582, 583.0, 583.1, 583.2, 583.4, 583.6, 583.7, 585, 586, 588.0, V420, V451, V56 |
| Cancer | 140, 141, 142, 143, 144, 145, 146, 147, 148, 149, 150, 151, 152, 153, 154, 155, 156, 157, 158, 159, 160, 161, 162, 163, 164, 165, 170, 171, 172, 174, 175, 176, 179, 180, 181, 182, 183, 184, 185, 186, 187, 188, 189, 190, 191, 192, 193, 194, 195, 200, 201, 202, 203, 204, 205, 206, 207, 208, 238.6 |
| Metastatic carcinoma | 196, 197, 198, 199 |
| **Abuse of alcohol and drugs** |  |
| Abuse of drugs | 304.0,304.1,304.2,304.3,304.4,304.5,304.6,304.7,304.8,304.9,305.2,305.3,305.4,305.5,305.6,305.7,305.8,305.9,292.0,969.6,965.01,292.82,292.83,292.84,292.89,292.9,292.11,292.12,292.2,648.3 |
| Abuse of alcohol | 305.0,303.0,303.9,291.0,291.1,291.2,291.3,291.4,291.5,291.8,291.9,571.0,571.1,571.2,571.3,425.5,535.3,357.5,265.2, V11.3,790.3,980.0 |
| Abuse of tobacco | 305.1,V158.2 |

Table B. **Estimation of the number of people aged over 15 years coinfected with HIV and HCV in Spain.**

| **Year** | **HIV-infected patients (No.) (*)** | **HCV-positive antibody (%) (‡)** | **HIV/HCV-coinfected patients (%)** (†) | **HIV/HCV-coinfected patients (No.)** (¥) | **HIV-monoinfected patients (No.)** (§) |
| --- | --- | --- | --- | --- | --- |
| **1997** | 106.483 | 69 | 69.8 | 74,353 | 32,130 |
| **1998** | 109.419 | 69 | 69.4 | 75,962 | 33,457 |
| **1999** | 111.281 | 69 | 68.9 | 76,689 | 34,592 |
| **2000** | 113.130 | 69 | 68.3 | 77,274 | 35,856 |
| **2001** | 114.824 | 69 | 66.7 | 76,605 | 38,219 |
| **2002** | 116.766 | 65 | 64.4 | 75,286 | 41,480 |
| **2003** | 118.964 | - | 62.1 | 73,905 | 45,059 |
| **2004** | 121.038 | 57.1 | 59.9 | 72,524 | 48,514 |
| **2005** | 123.069 | 58.5 | 57.7 | 71,113 | 51,956 |
| **2006** | 125.257 | 53.1 | 55.7 | 69,882 | 55,375 |
| **2007** | 127.615 | 50.8 | 53.8 | 68,719 | 58,896 |
| **2008** | 129.765 | 48.7 | 51.6 | 67,018 | 62,747 |
| **2009** | 131.888 | 47.7 | 49.2 | 64,992 | 66,896 |
| **2010** | 134.392 | 45.7 | 47.1 | 63,409 | 70,983 |
| **2011** | 136.747 | 42.6 | 45.4 | 62,126 | 74,621 |
| **2012** | 138.978 | 42.8 | 43.6 | 60,723 | 78,255 |
| **2013** | 141.052 | 42.6 | 41.9 | 59,163 | 81,889 |

(*) The estimation of the number of people living with HIV/AIDS in Spain was provided by the National Centre of Epidemiology (Instituto de Salud Carlos III, Madrid, Spain) [[1](#_ENREF_1)]. This estimation was made using the Estimation and Projection Package (EPP) and Spectrum software, both of which were developed by UNAIDS/WHO for estimating and projecting HIV prevalence at national level [[2](#_ENREF_2), [3](#_ENREF_3)].

(‡) The percentage of patients with HCV antibodies was collected from the “Asociación Médica VACH de Estudios Multicentricos (AMVACH)” (1999-2001) [[4](#_ENREF_4)], the “Grupo de Estudio de Sida” (GeSIDA) (2002) [[5](#_ENREF_5)], and the “Hospital survey of patients infected with HIV”, a second-generation surveillance system in people living with HIV coordinated by the National Centre of Epidemiology (2004-2013) [[6](#_ENREF_6), [7](#_ENREF_7)].

(†) The final estimation of the percentage of subjects coinfected with HIV and HCV in Spain was obtained from a regression model for imputing missing values and smoothing the numbers according to the temporal trend of the data.

(¥) The estimation of the number of subjects coinfected with HIV and HCV in Spain was the result of multiplying the number of individuals infected with HIV and the percentage of patients coinfected with HIV and HCV.

(§) The estimation of the number of HIV-monoinfected patients in Spain was the result of subtracting the number of patients coinfected with HIV and HCV from the number of individuals infected with HIV.

**REFERENCES:**

1. UN Joint Programme on HIV/AIDS. Global Report: UNAIDS Report on the Global AIDS Epidemic: 2010, December 2010, ISBN 978-92-9173-871-7, available at: <http://www.unhcr.org/refworld/docid/4cfca9c62.html> [accessed 2 December 2012]

2. Brown T, Bao L, Raftery AE, Salomon JA, Baggaley RF, Stover J*, et al.* Modelling HIV epidemics in the antiretroviral era: the UNAIDS Estimation and Projection package 2009. *Sex Transm Infect* 2010,**86 Suppl 2**:ii3-10.

3. Stover J. Projecting the demographic consequences of adult HIV prevalence trends: the Spectrum Projection Package. *Sex Transm Infect* 2004,**80 Suppl 1**:i14-18.

4. Roca B, Suarez I, Gonzalez J, Garrido M, de la Fuente B, Teira R*, et al.* Hepatitis C virus and human immunodeficiency virus coinfection in Spain. *J Infect* 2003,**47**:117-124.

5. González-García J, Navarro San Francisco C, E. C, Díez Romero C, Rodríguez-Navarro C, Zamora Vargas F*, et al.* Trends in HCV infection, liver disease severity and specific treatment modalities among HIV-infected patients in Madrid, Spain. GESIDA 5707 study. In: *19th Conference on Retroviruses and Opportunistic Infections (CROI 2012)*. Seattle, WA, USA; March 5-8, 2012.

6. Centro Nacional de Epidemiología/Subdirección General de Promoción de la salud y Epidemiología-Plan Nacional sobre el Sida. Encuesta Hospitalaria de pacientes con VIH/sida. Resultados 2013. Análisis de la evolución 2000-2013. Madrid; 2014, available at: http://www.msssi.gob.es/ciudadanos/enfLesiones/enfTransmisibles/sida/vigilancia/InformeEncuestaHospitalaria2014.pdf.

7. Diez M, Diaz A, Garriga C, Pons M, Ten A, Marcos H*, et al.* A low-cost, sustainable, second generation system for surveillance of people living with HIV in Spain: 10-year trends in behavioural and clinical indicators, 2002 to 2011. *Euro Surveill* 2014,**19**:pii: 20805.

Table C. **Incidence of stroke diagnosed (events per 10,000 person-years) in Spain (1997-2013) stratified by calendar period.**

|  | **Hemorrhagic stroke** | | | | | **Ischemic stroke** | | | | |
| --- | --- | --- | --- | --- | --- | --- | --- | --- | --- | --- |
|  | **HIV-monoinfected** | | **HIV/HCV-coinfected** | |  | **HIV-monoinfected** | | **HIV/HCV-coinfected** | |  |
|  | **No.** | **Rate (95%CI)** | **No.** | **Rate (95%CI)** | **^(a)^ p value** | **No.** | **Rate (95%CI)** | **No.** | **Rate (95%CI)** | **^(a)^ p value** |
| **Periods** |  |  |  |  |  |  |  |  |  |  |
| Whole period | 802 | 8.8 (8.2; 9.4) | 470 | 4 (3.6; 4.3) | <0.001 | 2122 | 23.3 (22.3; 24.3) | 801 | 6.7 (6.3; 7.2) | <0.001 |
| 1997-1999 | 158 | 15.8 (13.3; 18.2) | 29 | 1.3 (0.8; 1.7) | <0.001 | 274 | 27.4 (24.1; 30.6) | 41 | 1.8 (1.3; 2.4) | <0.001 |
| 2000-2003 | 178 | 11.1 (9.5; 12.7) | 85 | 2.8 (2.2; 3.4) | <0.001 | 422 | 26.3 (23.8; 28.8) | 117 | 3.9 (3.2; 4.6) | <0.001 |
| 2004-2007 | 185 | 8.6 (7.4; 9.9) | 148 | 5.2 (4.4; 6.1) | <0.001 | 483 | 22.5 (20.5; 24.5) | 192 | 6.8 (5.8; 7.8) | <0.001 |
| 2008-2013 | 281 | 6.5 (5.7; 7.2) | 208 | 5.5 (4.8; 6.3) | 0.083 | 943 | 21.7 (20.3; 23) | 451 | 11.9 (10.8; 13.1) | <0.001 |
| **^(b)^ p values** |  | *<0.001* |  | *<0.001* |  |  | *<0.001* |  | *<0.001* |  |

Values were expressed as absolute count and rate (95% confidence interval [95% CI]).

^(a)^ p values were calculated using the Extended Mantel-Haenszel chi-square test for linear trend.

Abbreviations: HCV, hepatitis C virus; HIV, human immunodeficiency virus.

Table D. **Case fatality rate of stroke (percentage) in Spain (1997-2013) stratified by calendar period.**

|  | **Hemorrhagic stroke** | | | | | **Ischemic stroke** | | | | |
| --- | --- | --- | --- | --- | --- | --- | --- | --- | --- | --- |
|  | **HIV-monoinfected** | | **HIV/HCV-coinfected** | |  | **HIV-monoinfected** | | **HIV/HCV-coinfected** | |  |
|  | **No.** | **Rate (95%CI)** | **No.** | **Rate (95%CI)** | **^(a)^ p value** | **No.** | **Rate (95%CI)** | **No.** | **Rate (95%CI)** | **^(a)^ p value** |
| **Periods** |  |  |  |  |  |  |  |  |  |  |
| Whole period | 318 | 39.6 (36.3; 43.1) | 215 | 45.7 (41.2; 50.4) | 0.039 | 263 | 12.4 (11.1; 13.9) | 106 | 13.2 (11.0; 15.8) | 0.584 |
| 1997-1999 | 75 | 47.4 (39.5; 55.5) | 12 | 41.4 (24.1; 60.9) | 0.688 | 39 | 14.2 (10.4; 19.1) | 7 | 17.1 (7.7; 32.65 | 0.808 |
| 2000-2003 | 81 | 45.5 (38.1; 53.1) | 43 | 50.6 (39.6; 61.5) | 0.522 | 60 | 14.2 (11.1; 18.0) | 17 | 14.5 (8.9; 22.5) | 0.999 |
| 2004-2007 | 76 | 41.1 (33.9; 48.5) | 67 | 45.3 (37.1; 53.6) | 0.512 | 61 | 12.6 (9.8; 1.0) | 32 | 16.7 (11.8; 22.8) | 0.212 |
| 2008-2013 | 86 | 30.6 (25.3; 36.4) | 93 | 44.7 (37.9; 51.7) | 0.002 | 103 | 10.9 (9.0; 13.1) | 50 | 11.09 (8.4; 14.4) | 0.999 |
| **^(b)^ p values** |  | *0.010* |  | *0.784* |  |  | *0.082* |  | *0.133* |  |

Values were expressed as absolute count and rate (95% confidence interval [95% CI]).

(a) p values were calculated using the exact confidence intervals for incidence.

(b) p values were calculated using the Extended Mantel-Haenszel chi-square test for linear trend.

Abbreviations: HCV, hepatitis C virus; HIV, human immunodeficiency virus.

Table E. **Epidemiological and clinical characteristics of patients with a hospital admission and diagnosis of stroke from 1997 to 2013 in Spain: non–HIV-infected vs HIV-infected.**

|  | **Non–HIV-infected** | **HIV-infected** | ***P* value** |
| --- | --- | --- | --- |
| **No. of patients** | 1759168 | 4091 |  |
| **Gender (male), No. (%)** | 940170 (53.4) | 3250 (79.4) | <0.001 |
| **Age (years), median (IQR)** | 75 (16) | 45 (15) | <0.001 |
| **Arterial hypertension, No. (%)** | 863335 (49.1) | 708 (17.3) | <0.001 |
| **Diabetes mellitus without complications, No. (%)** | 416637 (23.7) | 366 (8.9) | <0.001 |
| **Diabetes mellitus with complications, No. (%)** | 45648 (2.6) | 52 (1.3) | <0.001 |
| **Renal disease, No. (%)** | 111883 (6.4) | 195 (4.8) | <0.001 |
| ***Charlson comorbidity index*, median (IQR)** | 2 (2) | 1 (2) | <0.001 |
| **Substance use around admission date, No. (%)** |  |  |  |
| Illicit drugs | 205452 (11.7) | 1844 (45.1) | <0.001 |
| Alcohol | 23301 (1.3) | 151 (3.7) | <0.001 |
| Tobacco | 184234 (10.5) | 1093 (26.7) | <0.001 |
| **Conditions influencing health status, No. (%)** |  |  | <0.001 |
| Surgical conditions (V42, V45) | 187670 (10.7) | 161 (3.9) | <0.001 |
| Trauma (E880* to E929*, E950 to E999*) | 38404 (2.2) | 109 (2.7) | 0.04 |
| **Diagnoses related to stroke, No. (%)** |  |  |  |
| Hemorrhagic stroke (ICD-9 430-432) | 298482 (17) | 1272 (31.1) | <0.001 |
| Subarachnoid hemorrhage (ICD-9 430) | 55880 (3.2) | 280 (6.8) | <0.001 |
| Intracerebral hemorrhage (ICD-9 431) | 196102 (11.1) | 756 (18.5) | <0.001 |
| Other and unspecified intracranial hemorrhage (ICD-9 432) | 57404 (3.3) | 288 (7) | <0.001 |
| Ischemic stroke (ICD-9 433-437) | 1497387 (85.1) | 2923 (71.4) | <0.001 |
| Occlusion and stenosis of precerebral arteries (ICD-9 433) | 278146 (15.8) | 385 (9.4) | <0.001 |
| Occlusion of cerebral arteries (ICD-9 434) | 740903 (42.1) | 1476 (36.1) | <0.001 |
| Transient cerebral ischemia (ICD-9 435) | 244457 (13.9) | 319 (7.8) | <0.001 |
| Acute but ill-defined cerebrovascular disease (ICD-9 436) | 101230 (5.8) | 159 (3.9) | <0.001 |
| Other and ill-defined cerebrovascular disease (ICD-9 437) | 323054 (18.4) | 816 (19.9) | 0.01 |
| **Length of hospital stay (days), median (IQR)** | 8 (10) | 11 (16) | <0.001 |
| **Sequelae of stroke, No. (%)** |  |  |  |
| Late effects of cerebrovascular disease (ICD-9 438) | 80943 (4.6) | 122 (3) | <0.001 |

*P* values were calculated using the chi-square test and Mann-Whitney test.

Abbreviations: HCV, hepatitis C virus; HIV, human immunodeficiency virus.

Table F. **Incidence of stroke diagnosed (events per 10,000 person-years) in Spain (1997-2013) stratified by calendar period: non–HIV-infected vs HIV-infected.**

|  | **Hemorrhagic stroke** | | | | | **Ischemic stroke** | | | | |
| --- | --- | --- | --- | --- | --- | --- | --- | --- | --- | --- |
|  | **Non–HIV-infected** | | **HIV-infected** | |  | **Non–HIV-infected** | | **HIV-infected** | |  |
|  | **No.** | **Rate (95%CI)** | **No.** | **Rate (95%CI)** | **^(a)^ p value** | **No.** | **Rate (95%CI)** | **No.** | **Rate (95%CI)** | **^(a)^ p value** |
| **Periods** |  |  |  |  |  |  |  |  |  |  |
| Whole period | 298482 | 4.1 (4; 4.1) | 1272 | 6.1 (5.7; 6.4) | <0.001 | 1497387 | 20.4 (20.3; 20.4) | 2923 | 13.9 (13.4; 14.4) | <0.001 |
| 1997-1999 | 44341 | 3.7 (3.7; 3.8) | 187 | 5.7 (4.9; 6.5) | <0.001 | 222831 | 18.7 (18.6; 18.8) | 315 | 9.6 (8.6; 10.7) | <0.001 |
| 2000-2003 | 66446 | 4 (4; 4.1) | 263 | 5.7 (5; 6.4) | <0.001 | 332409 | 20.2 (20.2; 20.3) | 539 | 11.6 (10.6; 12.6) | <0.001 |
| 2004-2007 | 71666 | 4.1 (4.1; 4.1) | 333 | 6.7 (6; 7.4) | <0.001 | 350847 | 20 (20; 20.1) | 675 | 13.6 (12.6; 14.6) | <0.001 |
| 2008-2013 | 116029 | 4.2 (4.2; 4.2) | 489 | 6 (5.5; 6.5) | <0.001 | 591300 | 21.4 (21.3; 21.4) | 1394 | 17.2 (16.2; 18.1) | <0.001 |
| **^(b)^ p values** |  | *<0.001* |  | *0.3477* |  |  | *<0.001* |  | *<0.001* |  |

Values were expressed as absolute count and rate (95% confidence interval [95% CI]).

^(a)^ p values were calculated using the Extended Mantel-Haenszel chi-square test for linear trend.

Abbreviations: HCV, hepatitis C virus; HIV, human immunodeficiency virus.

Table G. **Case fatality rate of stroke (percentage) in Spain (1997-2013) stratified by calendar period: non–HIV-infected vs HIV-infected.**

|  | **Hemorrhagic stroke** | | | | | **Ischemic stroke** | | | | |
| --- | --- | --- | --- | --- | --- | --- | --- | --- | --- | --- |
|  | **Non–HIV-infected** | | **HIV-infected** | |  | **Non–HIV-infected** | | **HIV-infected** | |  |
|  | **No.** | **Rate (95%CI)** | **No.** | **Rate (95%CI)** | **^(a)^ p-value** | **No.** | **Rate (95%CI)** | **No.** | **Rate (95%CI)** | **^(a)^ p value** |
| **Periods** |  |  |  |  |  |  |  |  |  |  |
| Whole period | 85892 | 28.78 (28.61; 28.94) | 533 | 41.9 (39.18; 44.67) | <0.001 | 160177 | 10.7 (10.65; 10.75) | 369 | 12.62 (11.45; 13.9) | <0.001 |
| 1997-1999 | 12725 | 28.7 (28.28; 29.12) | 87 | 46.52 (39.26; 53.94) | <0.001 | 26306 | 11.81 (11.67; 11.94) | 46 | 14.6 (10.99; 19.1) | <0.001 |
| 2000-2003 | 19917 | 29.97 (29.63; 30.32) | 124 | 47.15 (41.01; 53.37) | <0.001 | 37518 | 11.29 (11.18; 11.39) | 77 | 14.29 (11.5; 17.59) | <0.001 |
| 2004-2007 | 21292 | 29.71 (29.38; 30.05) | 143 | 42.94 (37.59; 48.46) | <0.001 | 38024 | 10.84 (10.74; 10.94) | 93 | 13.78 (11.31; 16.66) | <0.001 |
| 2008-2013 | 31958 | 27.54 (27.29; 27.8) | 179 | 36.61 (32.36; 41.07) | <0.001 | 58329 | 9.86 (9.79; 9.94) | 153 | 10.98 (9.41; 12.76) | <0.001 |
| **^(b)^ p values** |  | *<0.001* |  | *<0.001* |  |  | *<0.001* |  | 0.0176 |  |

Values were expressed as absolute count and rate (95% confidence interval [95% CI]).

(a) p values were calculated using the exact confidence intervals for incidence.

(b) p values were calculated using the Extended Mantel-Haenszel chi-square test for linear trend.

Abbreviations: HCV, hepatitis C virus; HIV, human immunodeficiency virus.

Fig A. **Adjusted odds of death due to hemorrhagic and ischemic stroke in Spain (1997-2013) in HIV-infected patients in comparison with non–HIV-infected patients**.


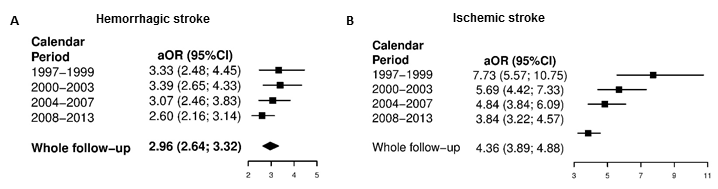


Abbreviations: aOR, adjusted odds ratio; 95%CI, 95% of confidence interval.
